# Impacts of offshore wind farms on the atmospheric environment over Taiwan Strait during an extreme weather typhoon event

**DOI:** 10.1038/s41598-022-04807-w

**Published:** 2022-01-17

**Authors:** Tsung-Yu Lee, Yu-Ting Wu, Mien-Tze Kueh, Chuan-Yao Lin, Yi-Ying Lin, Yang-Fan Sheng

**Affiliations:** 1 Department of Geography, National Taiwan Normal University, Taipei, Taiwan; 2 Department of Engineering Science, National Cheng Kung University, Tainan, Taiwan; 3 Research Center for Environmental Changes, Academia Sinica, Taipei, Taiwan

**Keywords:** Climate sciences, Environmental sciences

## Abstract

Wind is one of the cleanest renewable energy resources. Through the “Thousand Wind Turbines Project”, Taiwan is planning to increase the proportion of power generation from renewable energy and has set a target of 5.7 GW for offshore wind by 2025. The effects of future offshore wind farms (OWFs) over the Taiwan Strait on the atmospheric environment have not been evaluated. This study examined the potential effects of proposed OWFs on the atmospheric environment if the OWFs had existed during Tropical Storm Haitang (2017) by using Weather Research and Forecasting (WRF) model. A small set of ensemble simulations was conducted for studying the sensitivity of the ambient conditions in the region to the wind farm locations, the number and density of the turbines, and the initial time of simulations. Following the landfall and northward movement of Tropical Storm Haitang, a series of complex interactions between the typhoon circulation and the wind farm emerged, including small time slots of wake effect and mountain blocking effect. The combination of these rapidly changing OWFs-related effects contributed to a weak reduction in precipitation (− 1.08 mm) and hub-height wind speed (− 0.25 m s^−1^), as well as minimal warming near the surface (+ 0.13 °C) over southern Taiwan.

## Introduction

The global demand for renewable energy is increasing rapidly. According to a report published by the International Energy Agency (IEA)^1^, renewable energy is expected to increase by approximately 50%, and total installed solar and wind capacity will overtake both coal and gas to become the largest source of electricity generation globally in the next 5 years. Wind farms can generate clean electricity by extracting energy from the atmosphere. As wind flow passes through the wind turbine, wakes are generated downwind of the turbine, resulting in the reduction of wind speed, and the disturbed wind flow may affect local weather.

Wind farms can affect local circulation and regional weather; if sufficiently large, they may even alter the structure of weather systems like low-level jets (Larsén and Fischereit^2^). Using remote sensing, Christiansen and Hasager^3^ reported that as wind flow passed through large arrays of wind turbines, the mean wind speed was reduced by 8–9% and wake length could exceed 20 km. Wang and Prinn^4^ suggested that large-scale wind farms can increase global precipitation by 10% in some areas; however, the overall changes were not significant. The WRF model with the farm parameterization by Fitch et al.^5^ (WRF WFP hereafter) has been used extensively to study wind farm impacts. For example, Vautard et al.^6^ examined the effect of future potential European wind farms on regional climate by considering the current and a future scenario. They found modest changes, generally limited to the winter season, and the impacts remain much weaker than the natural variability. Siedersleben et al.^7^ evaluated the impacts of wind turbines on these meteorological parameters. Lauridsen and Ancell^8^ used an ensemble approach with WRF WFP to quantify the sensitivity of meteorological variables to the presence of wind farms. They show that the wind farm-induced perturbations to nonlocal mid-latitude cyclones are statistically significant, and the cyclone magnitude is dependent on the wind farm size and location relative to the midlatitude cyclone genesis region and track.

Additionally, experimental observations and numerical model findings revealed that atmospheric stability affected the wake and power efficiency of wind turbines^3,6,7,9–15^. Magnusson and Smedman^9^ observed the wake structure behind a wind turbine under various stability conditions. They found that the velocity deficit was a function of atmospheric stratification, with a larger velocity deficit observed under stable conditions. Hansen et al.^12^ showed that the more stable the atmospheric conditions, the larger the power deficit. Using a large eddy simulation model, Dörenkämper et al.^15^ found that wake effects were stronger in a stably stratified atmospheric boundary layer than under neutral or unstable conditions. In addition, they indicated that the distance of a wind farm to the coast was a crucial factor affecting power output. Recently, Pan et al.^16^ reported that hypothetical OWFs protected the coast from heavy precipitation during Hurricane Harvey. The horizontal divergence induced by the wind turbines caused a reduction in vertical motion and precipitation downstream of the farms. Al Fahel and Archer^17^ showed that the velocity deficit in wakes affected onshore precipitation and generated a divergence zone, which enhanced the vertical downward motion and suppressed precipitation. In addition, they reported that the effect of onshore precipitation was associated with the distance between OWFs and the coast.

Furthermore, the performance of WFP in the WRF model may depend on the horizontal and vertical resolution of the numerical model as well as the simulated atmospheric environment such as stability of atmospheric condition or other meteorological parameters^18,19^. However, a code bug was present in the WRF WFP (before the WRF version 4.2.1). Archer et al.^20^ indicated that the code bug has not been noticed because of the combination of the underestimation of TKE in the farm grid cell and the overestimation of TKE caused by the high value of the coefficient. They also claimed that the previous studies with the bug need to be revised to evaluate the impacts of wind farms. Larsén and Fischereit^2^ used the bug-fixed version of WRF to study the wind farm effects in the presence of low-level jets. They found that the value of the correction factor has a significant impact on the results.

**Figure 1:**
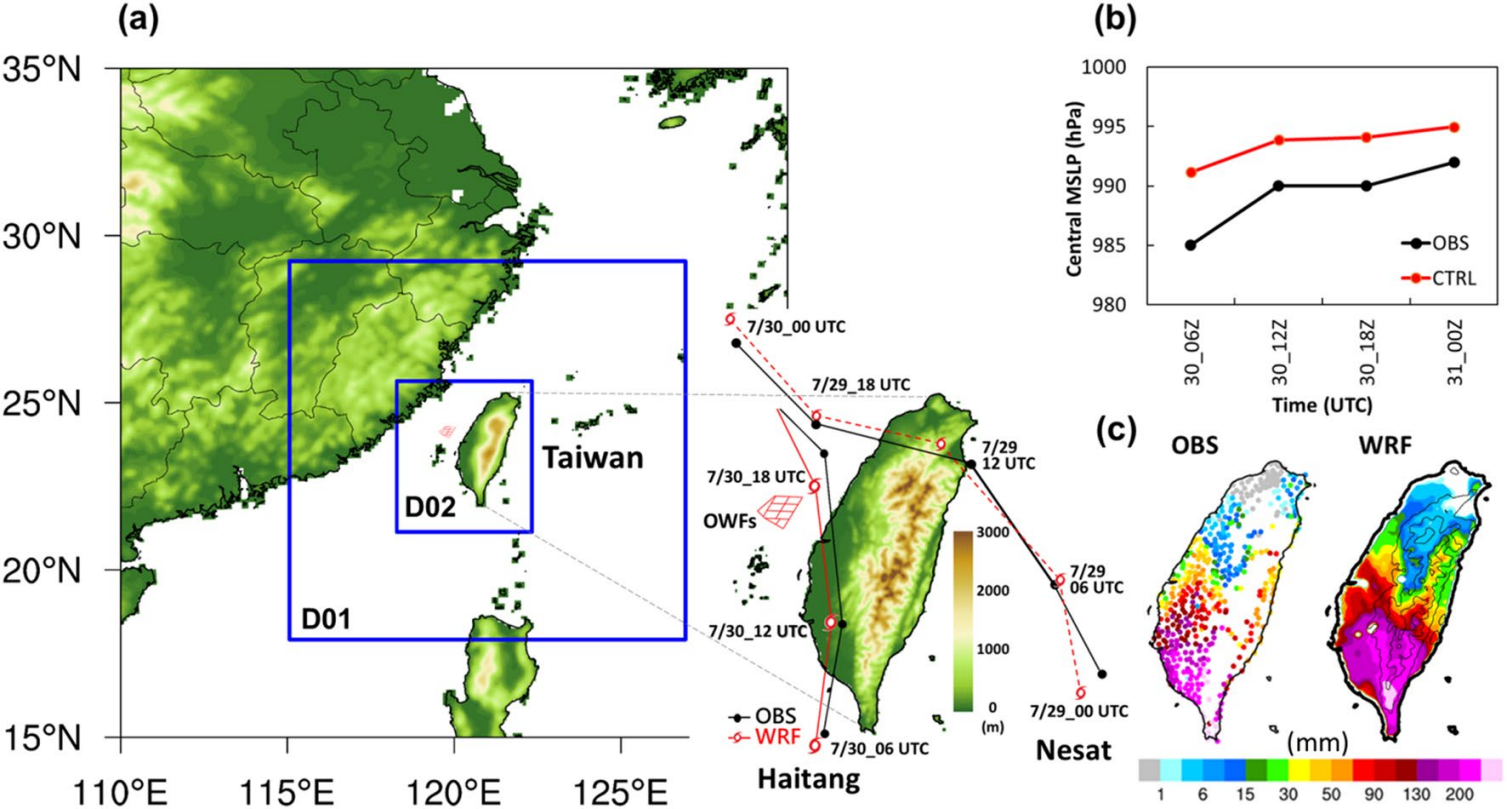
(**a**) Configuration of Weather Research and Forecasting model domain, topography, location of proposed offshore wind farms (OWFs) over Taiwan, and the comparisons of observed data (black line) and WRF model CTRL simulation (red line) of track. The simulated tracks of Typhoon Nesat and Tropical Storm Haitang are indicated by the dashed line and the solid line, respectively. OWFs location were indicated by the red area nearby western Taiwan. D01 and D02 indicate simulation domain 1 and domain 2, respectively. (**b**) Comparisons of observed (black line) and WRF model CTRL simulation (red line) in central minimum sea level pressure, and (**c**) observed and simulated accumulation precipitation over Taiwan during the period 06:00 UTC to 18:00 UTC 30 July, 2017. Maps and plots were produced using NCAR Command Language (NCL) version 6.6.2^37^.

Taiwan is located in the western North Pacific (Fig. 1a), which is one of the most active areas for tropical cyclones. On average, Taiwan experienced 4.8 typhoons per year between 1979 and 2016^21^. According to Taiwan’s “Four-year Wind Power Promotion Plan,” a goal of 5.7 GW before 2025 has been set for the capacity of OWFs (https://www.twtpo.org.tw/eng/offshore/directions.aspx). The number of wind turbines is expected to increase rapidly within the next few years. Once a tropical cyclone comes, its circulation could be altered by the OWFs and the coastal area would be influenced. To date, few studies have examined the effect of OWFs on local meteorological parameters and circulation over Taiwan, particularly extreme weather events. The objective of this paper is to assess the potential impacts of Taiwan’s proposed OWFs during a tropical cyclone by using the WRF model, which could be provided as a reference for the authorities in environmental impacts and management. Section “Model setup and data” describes the model configuration and the proposed OWFs. Section “Results” presents a detailed analysis of the potential effects of OWFs and the results of sensitivity simulation and discussion in section “Discussion”. Section “Conclusions” summarizes the results.

## Model setup and data

### Model configuration

In this study, we used the WRF model v4.2.2^22^ with Wind Farm Parameterization (WFP) proposed by Fitch et al.^5^ to investigate the effects of proposed OWFs on meteorological parameters over western Taiwan if the OWFs had existed during Tropical Storm Haitang (July 30–31, 2017). Archer et al.^20^ found that the treatment of TKE in the WRF WFP was incorrect and the value was too high. The correction factor is set to 0.25 following Archer et al.^20^. Simulations were performed using two nested domains with a horizontal grid spacing of 6 km (domain 1, 211 × 211 grid points) and 2 km (domain 2, 211 × 253 grid points), respectively (Fig. 1a). One-way nesting was employed in the simulation. The initial and boundary conditions were provided by the National Centers for Environmental Prediction/National Center for Atmospheric Research (NCEP/NCAR) and Global Data Assimilation System with a resolution of 0.25° at an interval of 6 h. The vertical layer consisted of 64 layers with 26 levels below 1 km and 4 levels within the rotor disk (i.e., 28–192 m), which had the lowest eta level of 0.996 (approximately 17 m above surface) and the highest terrain-following hydrostatic pressure of 10 hPa. The MODIS land use/land cover (LULC) and USGS Global Multi-resolution Terrain Elevation Data (GMTED2010, 30 arc seconds, ~ 1 km) were applied for the WRF model. The model was used to simulate 2 days—from 00:00 UTC (i.e. 00 Z) on July 29, 2017, to 00:00 UTC on July 31, 2017—to examine the effect of proposed OWFs during Tropical Storm Haitang. The first day was considered the spin-up time of the model, and the model output was hourly. In terms of physical parameterizations, the WRF single-moment 5-class microphysics scheme^23^ and land surface physics with the Noah Land Surface Model^24^ were adopted in the simulation. Furthermore, the Mellor–Yamada–Nakanishi–Niino 2.5-level planetary boundary-layer scheme^25^ was used for wind farm parameterization (WFP) in the WRF model. Fitch et al.^5^ developed WFP in the WRF model such that it represents a wind farm as an elevated momentum sink and an added turbulent kinetic energy (TKE) source. Based on the method reported by Blahak et al.^26^, Fitch et al.^5^ modeled the turbine drag by using the total fraction of kinetic energy extracted from the atmosphere by wind turbines, depending on thrust and power coefficients; additional details are given by Fitch et al.^5^. The aim of this study is not to simulate the track of typhoons perfectly. To make the track of the simulation with/without offshore wind farms consistent, the four-dimensional data assimilation (grid nudging) technique was used in the outer domain (i.e. domain 1). To examine the effects of proposed OWFs on the atmospheric environment during the study period, differences from the inner domains (i.e. domain 2) between the simulations with OWFs (denoted as “case 356-WTs”) and without OWFs (denoted as “case CTRL”) were presented in this study.

### Proposed OWFs

In Taiwan, the “Thousand Wind Turbines Project” was approved by the government in 2012. The Bureau of Energy, Ministry of Economic Affairs (MOEA), has been actively promoting wind power development. According to the “Four-Year Wind Power Promotion Plan,” the MOEA is planning to increase the proportion of power generated from renewable energy and has set a target of 5.7 GW for offshore wind by 2025. Most of the proposed OWFs (Fig. 1a) are located offshore of Changhua County, and they have a potential output capacity of 2400 MW, accounting for 62.6% of the total offshore wind power capacity. Moreover, many offshore wind turbines will be installed in the Taiwan Strait within the next few years.

In terms of the power capacity, if we assume that the type of wind turbine is the Vestas V164–8.0 MW^27^ in this study, then the total number of wind turbines required would be as high as 356. Therefore, we artificially installed 356 wind turbines in this area only in the inner domain (i.e. domain 2) of WRF model, following the offshore wind projects of Taiwan’s government. The location and layout of offshore wind farms in the model are shown in Fig. 1a,b, and at a distance of 40–70 km from the coastline of Taiwan. The turbine, with a 164-m rotor diameter (D), had a rated capacity of 8.0 MW and a hub height of 110 m above mean sea level. The cut-in and cut-out wind speeds were 4.0 and 25.0 m s^−1^, respectively. The inter-turbine spacing was 8 D in the east–west direction and 6.4–11.6 D in the south–north direction.

For the sensitivity analysis, five additional simulations with different inter-turbine spacing, OWF location and initial conditions were examined (Table 1). Case 711-WTs covered the same area but with double the density of wind turbines as in case 356-WTs, the inter-turbine spacing was 4 D in the east–west direction. Case 356-WTs_W was a case in which the OWFs in case 356-WTs were moved 20 km westward. Similarly, Case 356-WTs_E was a case in which the OWFs in case 356-WTs were moved 20 km eastward. Another two experiments by changing the initial time of simulations: case 356-WTs_2906Z and case 356-WTs_2912Z was the case in which the same configuration in case CTRL, but the initial time start 6 and 12 h after the case CTRL.

**Table 1:** Summary of simulation cases and the differences in accumulated precipitation, hub-height wind speed (WS), and 2-m temperature (T2) compared with that in CTRL over southern Taiwan (22.5–24.0° N, 119.5–121° E) for the period 12:00 UTC to 23:00 UTC on July 30, 2017.

Cases	No. of turbines	Initial time	12-h accu. rain (mm)	12-h rain diff. with CTRL (mm)	WS diff. with CTRL (m s^−1^)	T2 diff. with CTRL (°C)
CTRL	N/A	7/29_00Z	113.60			
356-WTs	356	7/29_00Z	112.52	− 1.08	− 0.25	0.13
711-WTs	711	7/29_00Z	113.69	0.09	− 0.24	0.33
356-WTs_E	356	7/29_00Z	113.70	0.10	− 0.25	0.11
356-WTs_W	356	7/29_00Z	113.26	− 0.34	− 0.21	0.37
356-WTs_2906Z	356	7/29_06Z	113.27	− 0.33	− 0.12	0.03
356-WTs_2912Z	356	7/29_12Z	112.39	− 1.21	− 0.02	0.08

## Results

### Model validation

To validate the performance of the WRF model, we examined the simulated track and MSLP of Tropical Storm Haitang, and precipitation during the study period against observations. The cyclone best track data and central minimum sea level pressure (MSLP) were obtained from the Japan Meteorological Agency (JMA) typhoon archive (http://www.jma.go.jp/jma/jma-eng/jma-center/rsmc-hp-pub-eg/besttrack.html). The hourly precipitation was provided by Central Weather Bureau (CWB), Taiwan from 522 rain gauge stations. Figure 1a,b show the comparison of track and MSLP, respectively, during July 30–31, 2017. Our simulated track was comparable to the observed track, whereas the intensity was underestimated during the study period (Fig. 1b). In particular, the simulated track agreed favorably with the observed track during the invasion period of the typhoon over Taiwan. The bias of the average track error was estimated to be lower than 24 km during 06:00–18:00 UTC on July 30, 2017. The simulated central MSLP (Fig. 1b) was slightly underestimated but generally followed the observed trend. The bias was generally lower than 5 hPa after landfall of the tropical storm in western Taiwan. The observed accumulated (06:00–18:00 UTC, 30 July) precipitation was 30–200 mm in southwest Taiwan and could be more than 300 mm over mountain ranges in southern Taiwan (Fig. 1c). The simulation reasonably reproduced and captured the spatial distribution of accumulated precipitation over Taiwan (Fig. 1c). A strong spatial correlation, with a correlation coefficient as high as 0.73, was observed between the simulated and observed data.

### Effects of OWFs on atmospheric parameters during the study period

**Figure 2:**
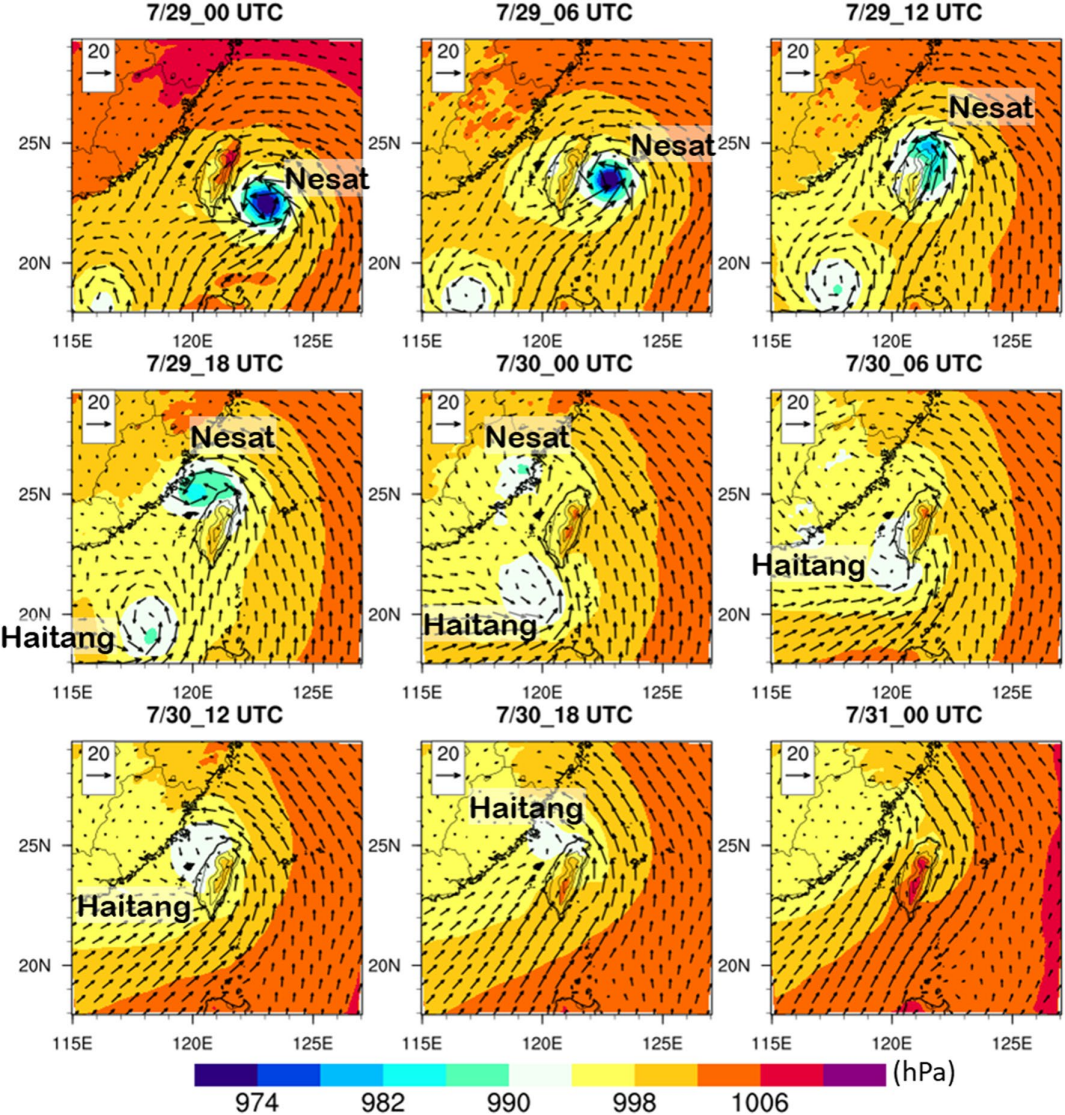
Sea level pressure (shaded, hPa) and 10-m horizontal wind (vector, m s^−1^) in the WRF model domain 1 (resolution is 6 km) during 29–31 July, 2017. Maps and plots were produced using NCAR Command Language (NCL) version 6.6.2^37^.

**Figure 3:**
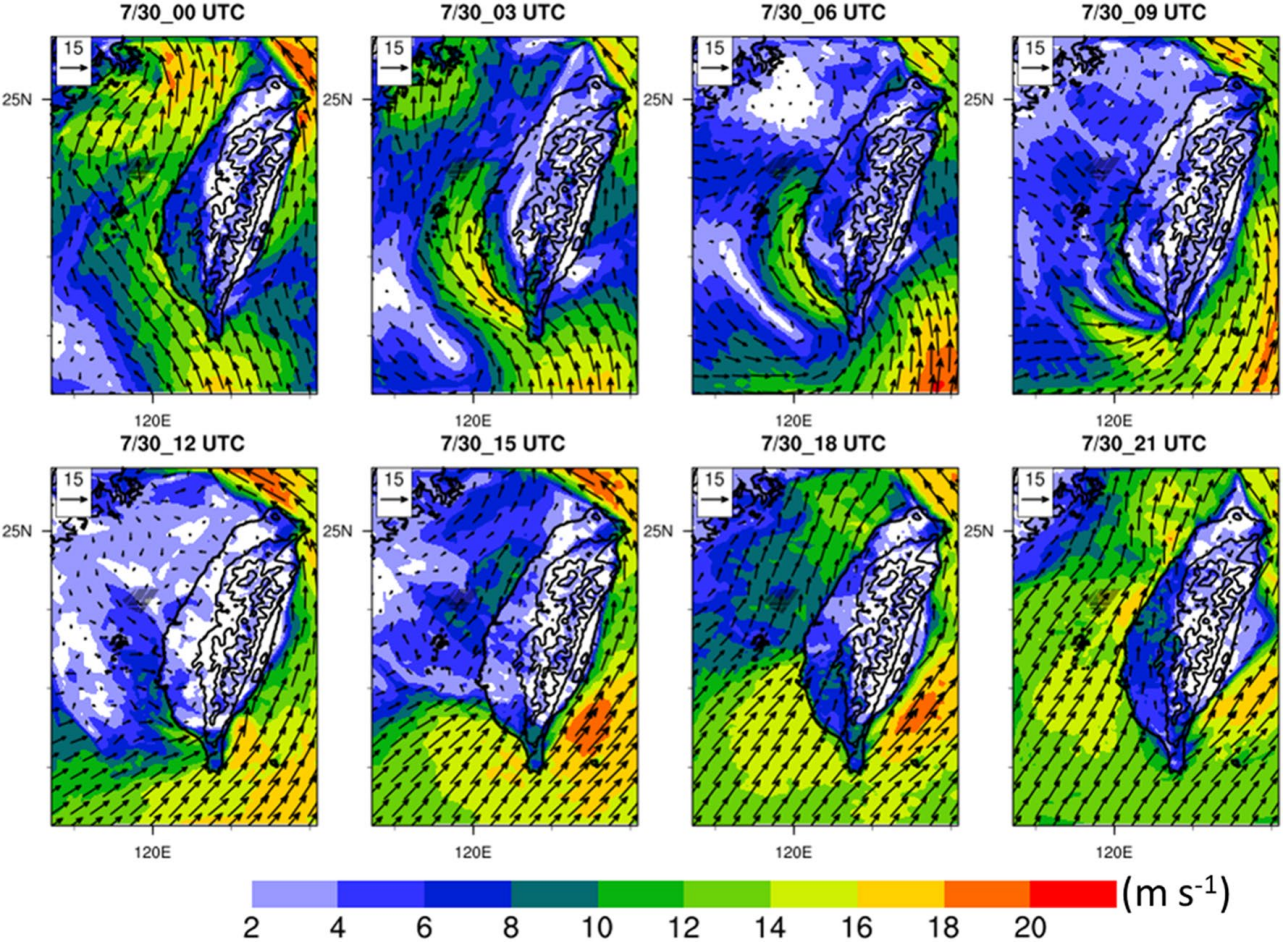
Hub-height (110 m) horizontal wind speed (shaded, m s^−1^) and horizontal wind (vector, m s^−1^) in the WRF model domain 2 (resolution is 2 km) during 00:00–21:00 UTC on July 30, 2017. The black contour lines indicate the elevation of Taiwan (m). The contour interval is 1000 m from 500 to 3500 m. Maps and plots were produced using NCAR Command Language (NCL) version 6.6.2^37^.

Although our simulation focused on the Tropical Strom Haitang, there was another typhoon Nesat coming from the ocean to the northeast of Taiwan on 29 July. In other words, Typhoon Nest was prior to Typhoon Haitang approaching Taiwan (Fig. 2) and the circulations were complex over Taiwan as it was hit by two tropical cyclones during 29–31 July (Fig. 2). Actually, a Fujiwhara effect was observed, i.e. these two tropical cyclones were in the proximity of one another, rotating cyclonically around a common center, and their tracks eventually interfered with each other. Typhoon Nesat made landfall on the northeastern coast of Taiwan at 11:00 UTC on July 29, 2017 (Fig. 2). Concurrently, Tropical Storm Haitang was located in the South China Sea and gradually moved toward southwestern Taiwan. As Typhoon Nesat made landfall over China (at 00:00 UTC 30 July in Fig. 2), its intensity was dramatically weakened. On the other hand, it induced a southwesterly flow over the Taiwan Strait (00:00–03:00 UTC 30 July in Fig. 3). A coupled low-pressure and a counter-clockwise rotation area can be seen around Taiwan, resulting from the interactions of these two cyclonic systems (Figs. 2, 3). In general, whether the OWFs impacted atmospheric parameters over western Taiwan or not was strongly related to the location of the typhoon and the corresponding ambient wind direction. In particular, the mean height of the Central Mountain Range (CMR) in Taiwan (Fig. 1a) is more than 2000 m^28^. The mountains do not only block but also uplift rainfall systems depending on the location of the cyclone and the direction of rain bands’ movement. Therefore, the strength of the effect depended on the variation of the cyclone track and the interaction of the wind field with the complex geographic structure. According to the track of Typhoon Nest on 29 July, the wind speed was weak over western Taiwan due to the blocking effect of the CMR (Fig. 2). Thus, in this study, we focused on the passage of Tropical Storm Haitang over Taiwan during 30–31 July 2017.

To examine the effects of proposed OWFs on the atmospheric environment during an extreme weather event, we conducted simulations with and without the inclusion of OWFs. Data showed that landfall of Tropical Storm Haitang occurred in Pingtung County in southern Taiwan at 08:40 UTC on July 30, 2017. Tropical Storm Haitang moved northward after 09:00 UTC and its low-pressure center was located in western Taiwan. However, strong typhoon’s cyclonic circulations interacted with the CMR mainly in the eastern of the island and resulted in a weak-wind region (less than 10 m s^−1^) on the lee side of the mountains over western Taiwan. It was noted that two different directions’ flow, northeasterly and northwesterly interacted near the OWFs between 09:00–12:00 UTC, on July 30 (Fig. 3). The flow over the Taiwan Strait was replaced with southwesterly between 15:00 and 21:00 UTC (Fig. 3) as Tropical Storm Haitang already moved to the north of OWFs. The wake effects^3,29–31^ could occur due to a reduction of wind speed downwind of OWFs on July 30. Siedersleben et al.^7^ used aircraft measurements and the WRF model to investigate all existing and planned wind farms in the North Sea. They reported that the effects of wakes on temperature and water vapor could propagate more than 100 km downwind under strong stable atmospheric conditions. In Taiwan, the distances of proposed OWFs from the west coast are 40–70 km. Thus, the atmospheric environment over western Taiwan is expected to be possibly affected by wind turbine wakes. Owing to the variation of wind field near the OWFs, the differences between the simulations with and without wind farms in other meteorological parameters (such as hub-height wind speed, 10-m divergence, atmospheric stability, and hub-height water vapor) over southern Taiwan were examined.

**Figure 4:**
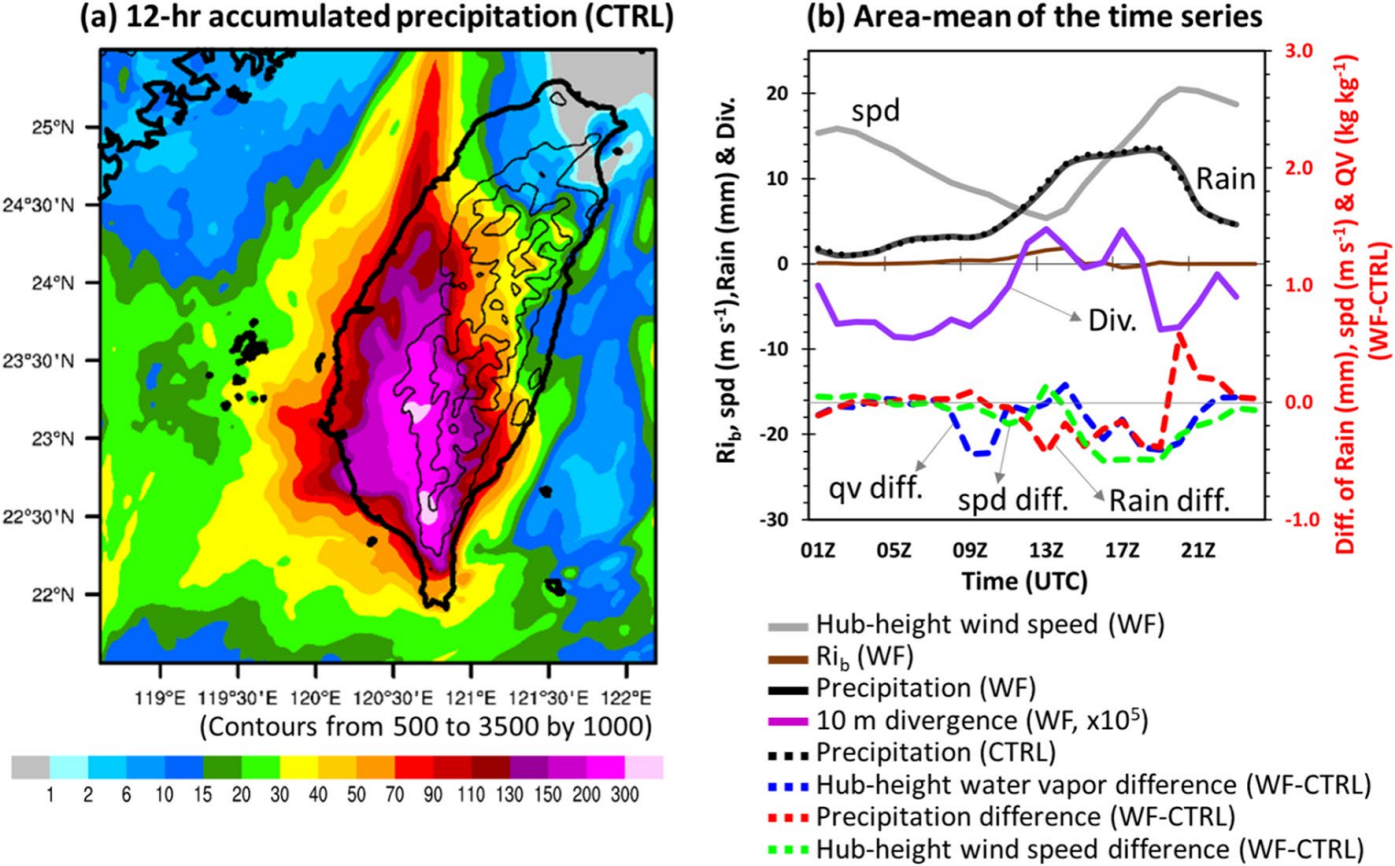
(**a**) 12-h accumulated precipitation (mm) in case CTRL over Taiwan during 12:00–23:00 UTC on July 30, 2017. Contours indicate the terrain height (m). (**b**) Area-mean (22.5–24° N, 119.5–121° E) of the time series of hub-height wind speed in case 356-WTs (denoted by the grey line, m s^−1^), 10-m divergence (purple line, s^−1^, left axis) and atmospheric stability (brown line, left axis) calculated from model levels 1 (~ 17 m) and 6 (~ 198 m) close to the rotor disk (28–192 m) in case 356-WTs (23.9–24.2° N, 120.0–120.1° E), and precipitation (black solid line, mm, left axis) of case 356-WTs and CTRL (black dashed line, mm, left axis), and the differences of precipitation (red dashed line, mm, right axis), hub-height wind speed (green dashed line, m s^−1^, right axis) and hub-height water vapor (blue dashed line, g kg^−1^, right axis) during 01:00–23:00 UTC on July 30, 2017. Maps and plots were produced using NCAR Command Language (NCL) version 6.6.2^37^.

Figure 4a shows the 12-h accumulated precipitation of case CTRL over Taiwan. Precipitation mainly occurs in southern Taiwan, and the difference with case 356-WTs is also found there (see the supplementary in Fig. S1). To quantify the reduction in precipitation downwind of OWFs in southern Taiwan, we particularly focused on the area ranging from 22.5° N to 24.0° N and 119.5° E to 121.0° E in this study. Simulation results indicated that the average reduction in 12-h accumulated precipitation over the focused area was approximately 1% (1.08 mm; Table 1), due to the values of positive and negative compensation. The maximum reduction was up to 60 mm over the mountain ranges in southern Taiwan and the maximum increase was up to 47 mm over the central plain of Taiwan. Furthermore, atmospheric stability may exert a strong effect on the vertical and lateral spread of the wake. Magnusson and Smedman^9^ determined the strength of the velocity deficit arose from the wake effect in terms of atmospheric stability. Abkar and Porté-Agel^32^ indicated that recovery from the wake was slower under stable conditions due to less extensive turbulent mixing. Herein, we calculated the bulk Richardson number (Rib) to estimate the atmospheric stability across the rotor disk using the data from two model levels 1 (17 m) and 6 (198 m):

1 $${\text{Ri}}_{{\text{b}}} = \frac{{\text{g}}}{{\overline{\uptheta }}}\frac{{\Delta \uptheta /\Delta {\text{z}} }}{{(\Delta {\text{U/}}\Delta {\text{z}})^{2} }}$$

where g is the gravitational acceleration, $\overline{\uptheta }$ is an average virtual potential temperature between the two model levels, $\Delta \uptheta$ and $\Delta {\text{U}}$ are the virtual potential temperature and wind speed differences, respectively, between these model levels. The two model levels are selected to approximately cover the rotor disk (28–192 m), and $\Delta {\text{z}}$ is the vertical distance between the two levels. This Rib calculation is similar to the research on wind farm by Dörenkämper et al.^15^. A bulk Richardson number of Rib > 0.1 is defined as stable, and near zero value is defined as neutral^33,34^.

Figure 4b shows the area-averaged time series of the hub-height wind speed, 10-m divergence, and precipitation over the focus area (22.5–24.0° N, 119.5–121° E), and the differences in hub-height water vapor, hub-height wind speed, and precipitation between 356-WTs and CTRL simulation. When the circulation center of the typhoon was located to the west of CMR, a weak-wind region formed on the lee side of the mountains under the condition of broad-scale cyclonic circulation. The area-mean hub-height wind speed (356-WTs) rapidly decreased from 8 to 5 m s^−1^ (from 10:00 UTC to 13:00 UTC, − 37.5%) due to typhoon circulation interacting with the complex terrain of CMR. With the presence of OWFs, the hub-height water vapor and wind speed began to decrease owing to the circulation being affected by the OWFs, and precipitation also decreased few hours later over southern Taiwan. The atmospheric stability (356-WTs) in the nearshore area (23.9–24.2° N, 120.0–120.1° E) in between the OWFs and land was neutral before landfall (at around 09:00 UTC on July 30, 2017) and then subsequently became stable in association with a development of broadscale weak near surface divergence (10:00–16:00 UTC). This stable condition reveals a development of nocturnal boundary layer (the local time offset is UTC + 08:00 here) in this area.

**Figure 5:**
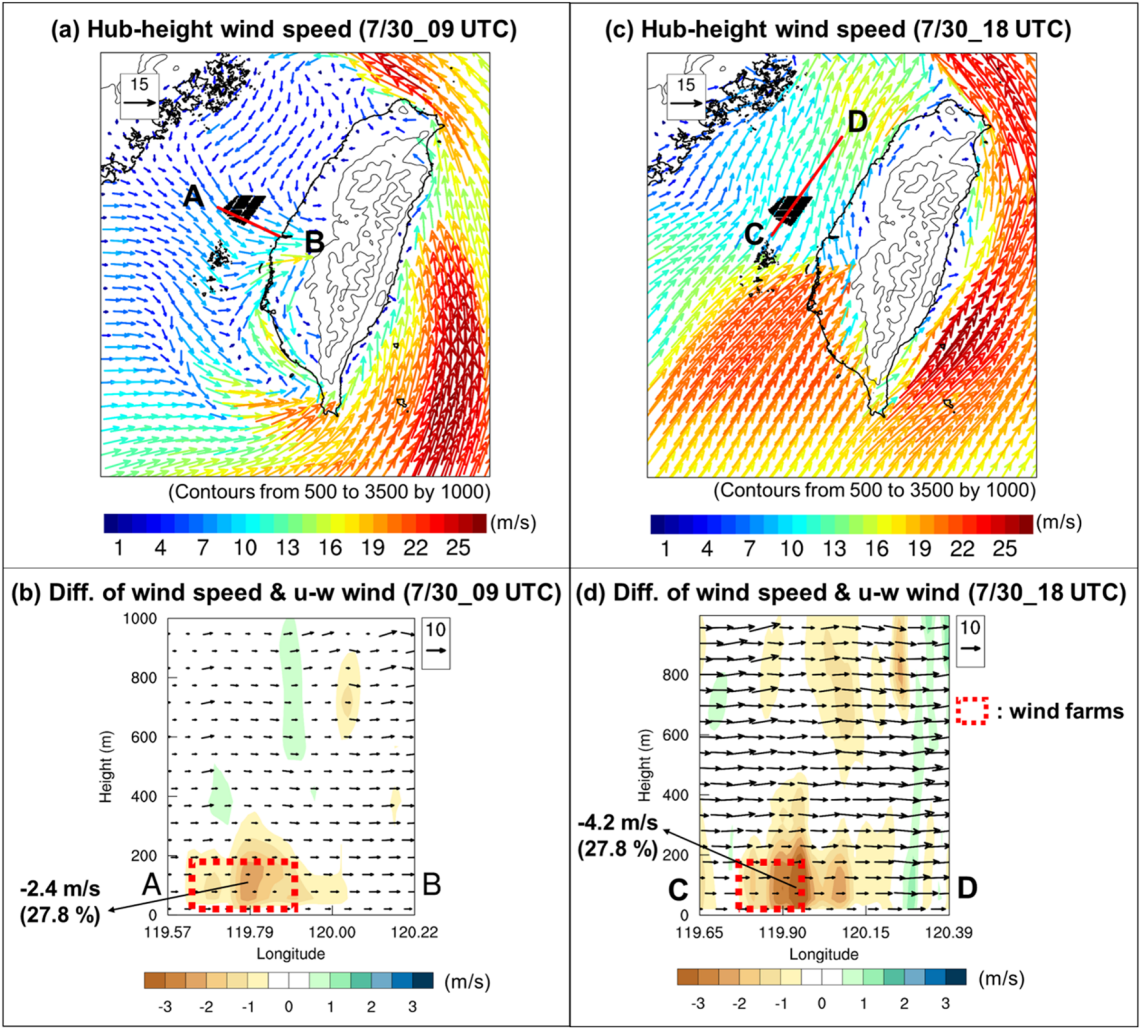
Vertical cross-sectional diagram of simulation (D02: resolution is 2 km) differences between case 356-WTs and CTRL along the line AB (CD) at 09:00 (18:00) UTC on July 30, 2017: (**a**) horizontal hub-height wind speed in case 356-WTs (vector, m s^−1^) at 09:00 UTC, (**b**) horizontal wind speed difference (shaded, m s^−1^) and u-w wind vectors in case 356-WTs (w-wind: × 10^2^, m s^−1^) at 09:00 UTC, (**c**) horizontal hub-height wind speed in case 356-WTs (vector, m s^−1^) at 18:00 UTC, (**d**) horizontal wind speed difference (shaded, m s^−1^) and u-w wind vectors in case 356-WTs (w-wind: × 10^2^, m s^−1^) at 18:00 UTC. Maps and plots were produced using NCAR Command Language (NCL) version 6.6.2^37^.

As Tropical Storm Haitang made landfall in southern Taiwan and toward the north (Fig. 1a), its cyclonic flow was weakened due to the blocking effect of the CMR. It was identified that the horizontal wind speed was over 20 m s^−1^ to the east of the CMR, however, at the lee side of the mountain, the wind speed was less than 10 m s^−1^ around the OWFs and the western plain of Taiwan (Fig. 3). Meanwhile, it can be seen that the typhoon’s circulation pass through the OWFs (Fig. 3) and the area mean precipitation is gradually increasing (Fig. 4b) at 12:00 UTC, 30 July. Few hours later, the area-averaged precipitation reached a high level of amount (> 10 mm) and persisted for about 6 h (Fig. 4b). As mentioned earlier, the typhoon kept moving northward and its circulation was complex and changing during the study period. It was not easy to appropriately examine the impact of the OWFs on the meteorological parameters at the downwind side. A northwesterly flow at 09:00 UTC (Fig. 5a), and a southwesterly flow at 18:00 UTC, 30 July (Fig. 5c) which passed over wind farm were employed to examine the occurrence of wake effect. Figure 5b,d illustrate the vertical cross-section in NW*–*SE (SW–NE) direction across the OWFs from A to B (C to D). Owing to the presence of the OWFs, a maximum wind speed decrease (~ 2.4 m s^−1^ and 4.2 m s^−1^) within the rotor area of the farm (near 119.79° E in Fig. 5b and 119.90° E in Fig. 5d). The influence of the OWFs in wind speed was approximately 28% and the wind farm wakes can be seen along these two cross-sections (Fig. 5b,d). Furthermore, the regions of wind farm wake varied with the corresponding wind directions which were associated with the varied circulations as shown in Fig. 5a,c. Although the ambient condition became stable along with weak near-surface divergence, thereby inhibiting the development of turbulent mixing, the wake effect in our case ceased rapidly within a few hours. This is in contrast with the results of Abkar and Porté-Agel^32^, primarily because of the rapidly changing wind directions in our case. Shortly, the wake effect only occurred within a few hours as the winds passed over the wind farms.

## Discussion

The previous section indicated the potential effects of OWFs on meteorological parameters by examining the simulation results of case 356-WTs. However, these features were inspected based on the comparison between a single set of perturbation experiments, namely, one experiment with OWFs compared with the control experiment without the OWFs. Ancell et al.^35^ pointed out that rapid perturbation growth can be a serious issue for studying the evolution of differences in model simulations by using perturbation experiments. They revealed that rapidly amplifying and propagating perturbations could result from unrealistic numerical noise and instability, and further suggested that the use of a set of ensemble experiments can mitigate this kind of issue. For example, Lauridsen and Ancell^8^ used an ensemble modeling approach with WFP of WRF model to successfully reveal wind farm-induced perturbations can evolve a couple of days or areas of thousands of kilometers, primarily by showing certain consistency among their ensemble members. Here, five additional simulations were performed to investigate the sensitivity of different initial times, OWF location, and inter-turbine spacing (Table 1). To examine whether there is rapid perturbation growth affecting the simulated results, we calculated the difference total energy (DTE) proposed by Zhang et al.^36^. The DTE per unit mass is defined by:

2 $$\frac{1}{2} \sum \left( {{U^{{\prime}2}}_{ijk} + {V^{{\prime}2}}_{ijk} + {\kappa T^{{\prime}2}}_{ijk} } \right)$$

where U′, V′, and T′ are the differences in wind and temperature between the simulations with and without WFP. κ is Cp/R (Cp is the specific heat capacity of air: 1004 J K^−1^ kg^−1^, R is 287 J K^−1^ kg^−1^), and i, j, and k mean the grid points in x, y, and σ. The vertical level from surface to top (10 hPa) over the full domain was calculated.

**Figure 6:**
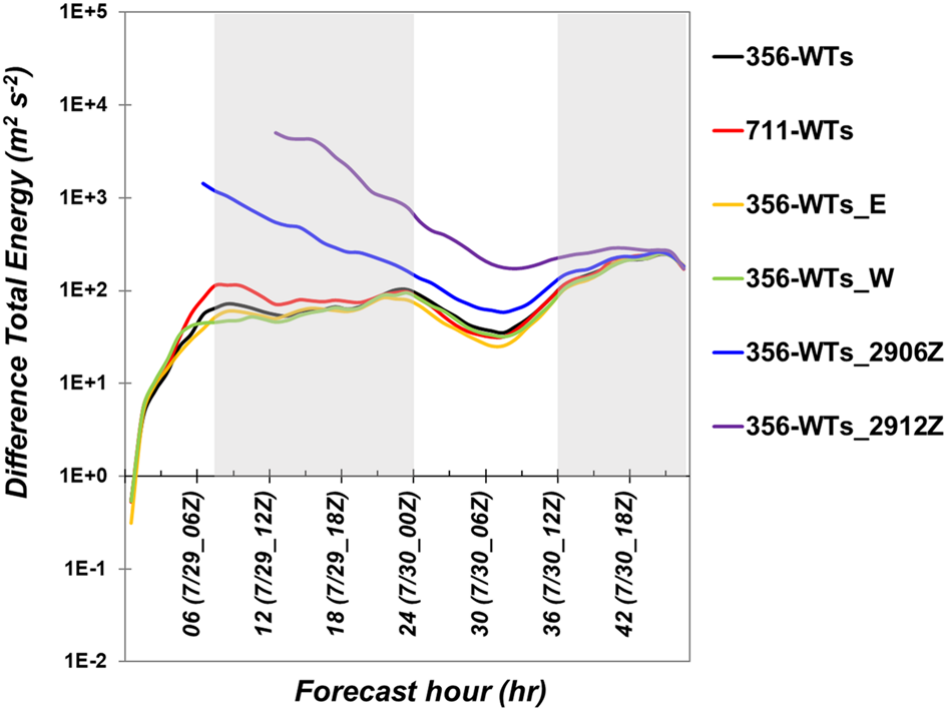
The evolution of estimated Difference Total Energy (DTE, m^2^ s^−2^) for all experiments. The black, red, yellow, green, blue and purple lines represent the case 356-WTs, case 711-WTs, case 356-WTs_E, case 365-WTs_W, case 356-WTs_2906Z and case 356-WTs_2912Z, respectively. Grey background indicates the two steadily stages. Plots were produced using NCAR Command Language (NCL) version 6.6.2^37^.

Figure 6 showed the evolution of DTEs for all experiments listed in Table 1. The DTEs existed two relatively “steady” stages and a break period in between. The two steady stages indicated the invasion period of two typhoons. For those experiments with the same initial time, the DTEs grew rapidly during the first 6 h, this is in expectation of the model spin-up process. The DTEs then underwent a spell of the steady stage until 00:00 UTC on July 30. The larger differences found in cases 356-WTs_2906Z and 356-WTs_2912Z were due to the different initial conditions. During the period when Typhoon Nesat was leaving and Tropical Storm Haitang was entering domain 2 (cf. Fig. 2, 00:00–12:00 UTC 30 July), DTEs in all experiments underwent decaying and rebounding tendency, indicating a break period. In other words, the DTEs was not growing up from the initial time all the way till the end of the simulation, and the emergence of the break period is suggestive of a reduction in the perturbation energy in the domain. Accordingly, we argue that the results during our focus period (12:00–23:00 UTC 30 July) did not be contaminated by the initial errors. Furthermore, the DTEs in all experiments showed the second steady stage consistently well, suggesting that there was no significant growing tendency of the perturbation errors after 36 h (Fig. 6). Thus, rapid perturbation growth was not a serious issue in our study as indicated by Ancell et al.^35^ and Zhang et al.^36^.

**Figure 7:**
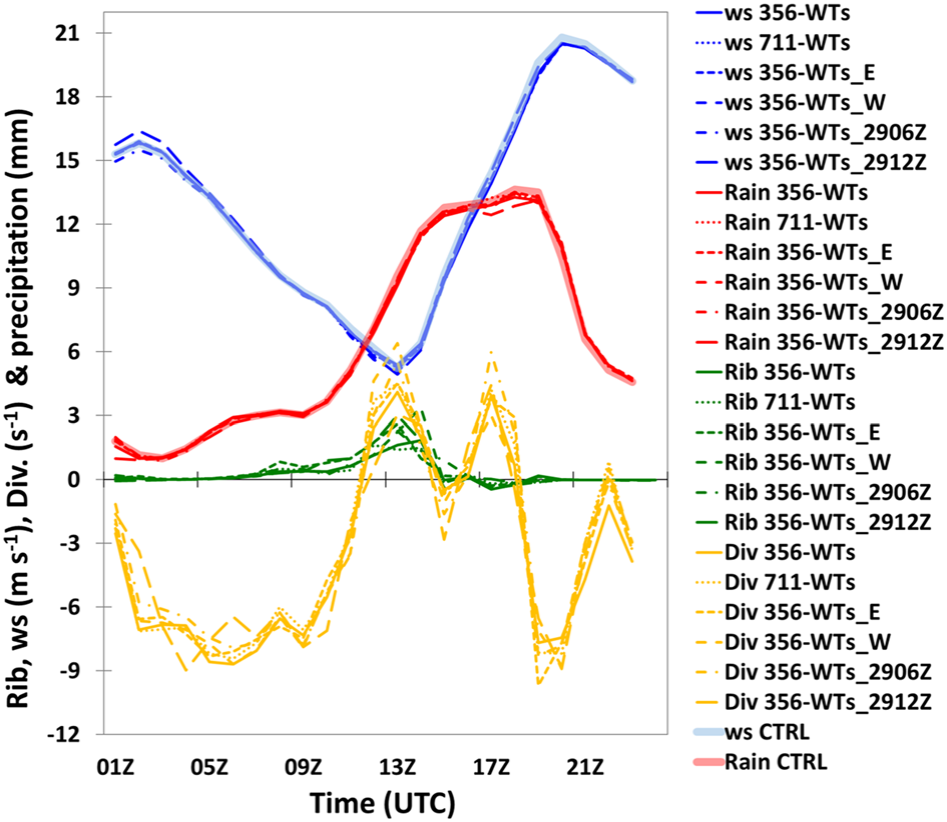
Same as Fig. 4b except for the all experiments over southern Taiwan during 01:00–23:00 UTC on July 30, 2017. Plots were produced using NCAR Command Language (NCL) version 6.6.2^37^.

Figure 7 shows the area-averaged time-series of the hub-height wind speed, precipitation, atmospheric condition, 10-m divergence, and the differences between the cases considered in the sensitivity simulations and CTRL, like that shown in Fig. 4. The sensitivity simulations in Fig. 7 were including the number of wind turbines (case 711-WTs, i.e., double number of the wind turbines), different locations of wind farms (case 356-WTs_E and case 356-WTs_W), and different initial times (6 or 12 h late than CTRL). The results indicated that all cases showed high similarity in the evolution of the atmospheric conditions (Ri_b_, Divergence), hub-height wind speed, and precipitation during 30 July (Fig. 7). Similar to the case 356-WTs, all other experiments indicated that the hub-height wind speed decreases rapidly during the break period (Fig. 6, 00:00–12:00 UTC), and the precipitation was gradually increasing during the second steady stage (Fig. 6) as a typhoon was approaching the OWFs (Fig. 4b). The development of the nearshore area of nocturnal stable condition across the layer of rotor disk were also well captured by all experiments.

**Figure 8:**
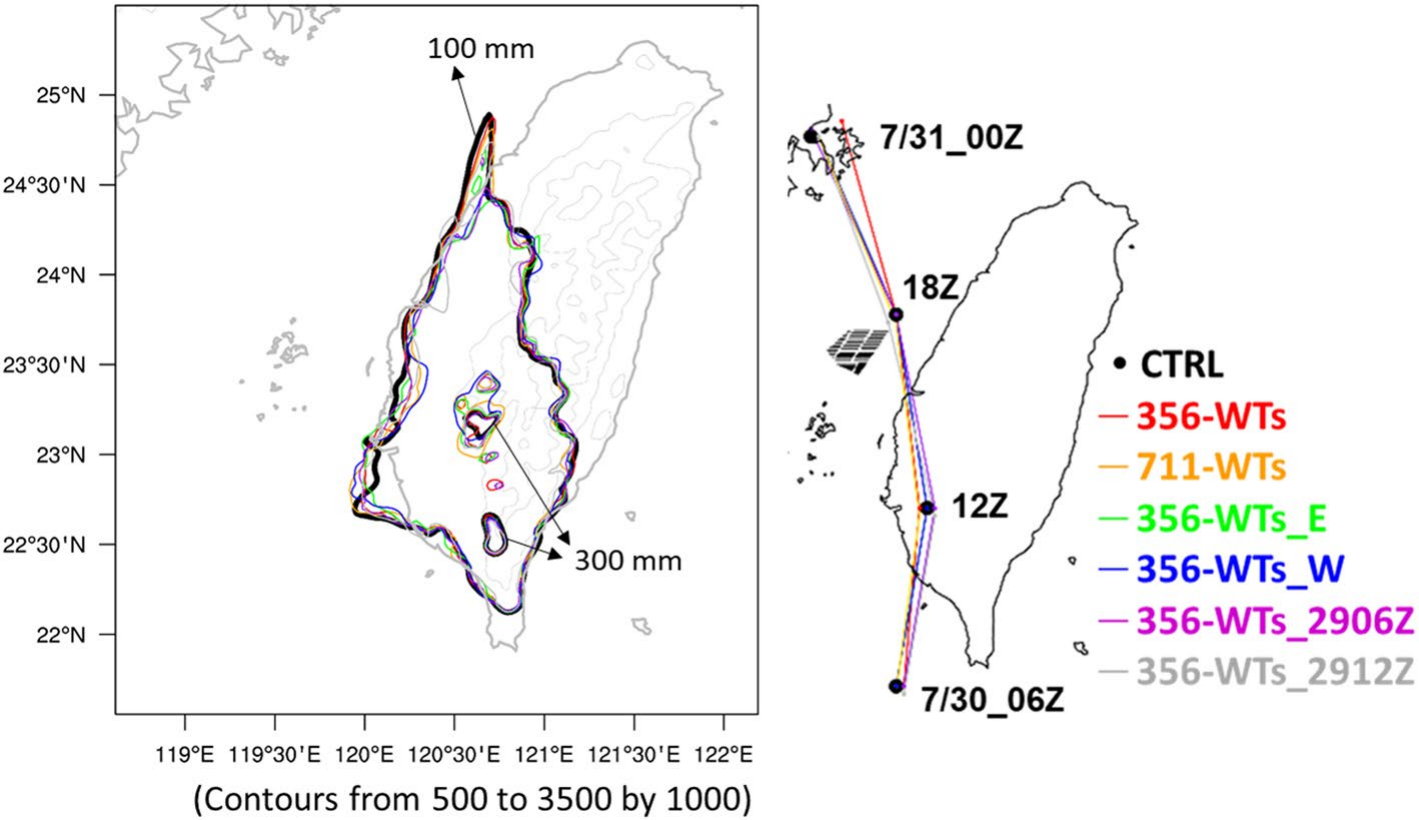
12-h accumulated precipitation over Taiwan during 12:00–23:00 UTC on July 30, 2017 and the track of Tropical Storm Haitang for all experiments. The outter and the inner contour lines indicate the precipitation of 100 mm and 300 mm, respectively. The grey contours indicate the terrain height (m). Maps and plots were produced using NCAR Command Language (NCL) version 6.6.2^37^.

To quantify precisely the impacts of OWFs on the environment for the design experiments, the time-averaged of the differences of winds, precipitations, and temperatures in the second steady stage (12:00–23:00 UTC on July 30, 2017) was calculated (Table 1). There was a good consistency of wind speed deficit and surface temperature warming at downwind of wind farms in all experiments. A maximum reduction in wind speed was 0.25 m s^−1^ while the maximum temperature increase was 0.37 °C in these six experiments (Table 1). In terms of precipitation, four out of six cases consistently showed a reduction related to the different initial conditions and the location of OWFs away from the shore. On the contrary, the precipitation increase was shown in the case of double the number of wind turbines and the location closer to the shore (i.e. 711-WTs and 356-WTs_E). The increase or decrease trends in precipitation among these experiments may come from the typhoon locations or some slight differences in the typhoon’s rain-bands. Figure 8 illustrats the 12-h accumulated precipitation and the typhoon tracks for all experiments during 12:00–23:00 UTC on July 30, 2017. The spatial distribution of accumulated precipitation was highlighted in 100 mm (outer contour) and 300 mm (inner contour). The distribution of accumulated rainfall presented a similar pattern among the experiments. There are, however, slight differences existed in the location of heavy rainfall. This may be attributed to the slight simulation differences in the Typhoon track.

**Figure 9:**
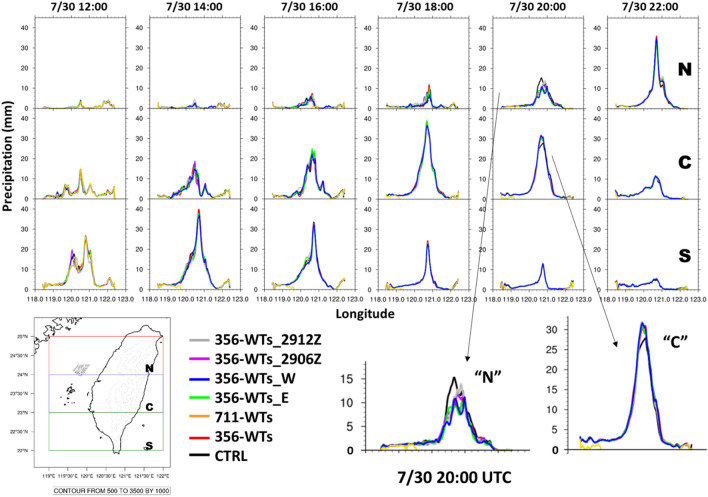
The evolution of hourly precipitation over three regions for all experiments during 12:00–22:00 UTC on July 30, 2017. North, central and south region denoted by “N”, “C”, and “S”, respectively. Maps and plots were produced using NCAR Command Language (NCL) version 6.6.2^37^.

The difference in the propagation of the rain bands can be another reason. Figure 9 presents the evolution of hourly precipitation (Fig. 9) from the south (denoted by S) to the north (denoted by N) in all experiments. As indicated in Fig. 8, the typhoon was gradually approaching Taiwan and passing through the OWFs from the S to N during our study period. Compared to the CTRL simulation, a time-lag of peak precipitation could be observed in the cases with wind farm simulations at 20:00 UTC. At this moment, the typhoon center was located at the north of the wind farms (Fig. 8) and precipitation at its downwind area was affected by the typhoon’s circulation. It was noted that the peak precipitation in all experiments with wind farm was higher than the CTRL at region C while it was lower than the CTRL at region N. The result suggested that the rain-band in CTRL was propagating faster than others at the time, resulting in differences in precipitation. The existed OWFs could be considered as a barrier or topographic. At the downwind area of OWFs, typhoon circulations could be blocked and perturbed. Even though the difference of area mean precipitation was not significant during our focus period due to the cancellation between positive and negative values over western Taiwan (Supplement S1). The consistency between these experiments still might be worth considering. Shortly, sensitivity results supported that OWFs impacts on the atmospheric environment should be considered including the density/size of OWFs but also the location of OWFs.

## Conclusions

This study evaluated the potential effects of proposed OWFs on the atmospheric environment when an extreme weather event, Tropical Storm Haitang as an example, occurred over Taiwan. The bug-fixed version of WRF model (version 4.2.2) with a WFP scheme was employed in this study. According to the energy policy of the Bureau of Energy, MOEA, the proposed OWFs with 356 wind turbines (case 356-WTs), were implemented in the model.

Compared with observation, the control simulation without OWFs discovered an MSLP that was slightly underestimated but generally exhibited the same trend. The WRF model could reproduce the spatial distribution of accumulated precipitation, with the corresponding correlation coefficient at 0.73. We conducted a small set of ensemble simulations for studying the sensitivity of the ambient conditions in the region to the wind farm locations, number and density of the turbines, and the initial time of simulations. During the period when Tropical Storm Haitang made landfall in southern Taiwan and moved northward, a series of complex interactions between the typhoon circulation and the wind farm were found, including small time slots of wake effect and blocking effect. The combination of these rapidly changing OWFs-related effects contributed to the changes in the ambient conditions, specifically the deficit of precipitation, the reduction in near surface wind speeds as well as the warming of near surface temperature.

## Supplementary Information

Supplementary Figure S1.
