# Supplementary material for: Impacts of offshore wind farms on the atmospheric environment over Taiwan Strait during an extreme weather typhoon event
Source: Sci Rep. 2022 Jan 17;12:823. doi: 10.1038/s41598-022-04807-w (PMC8763917; doi:10.1038/s41598-022-04807-w)
Supplement: Supplementary file 1 — Supplementary Figure S1. [file 41598_2022_4807_MOESM1_ESM.docx]

**Supplements for:**

Impacts of Offshore Wind Farms on the Atmospheric Environment over Taiwan Strait During an Extreme Weather Typhoon Event

**Tsung-Yu Lee^1^, Yu-Ting Wu^2^, Mien-Tze Kueh^3^, Chuan-Yao Lin^3,^**^*^**, Yi-Ying Lin^3^, and Yang-Fan Sheng^3^**

^1^Department of Geography, National Taiwan Normal University, Taipei, Taiwan.

^2^Department of Engineering Science, National Cheng Kung University, Tainan, Taiwan.

^3^Research Center for Environmental Changes, Academia Sinica, Taipei, Taiwan.

*Chuan-Yao Lin (yao435@rcec.sinica.edu.tw)

This file contains supplementary figure:

Figure S1


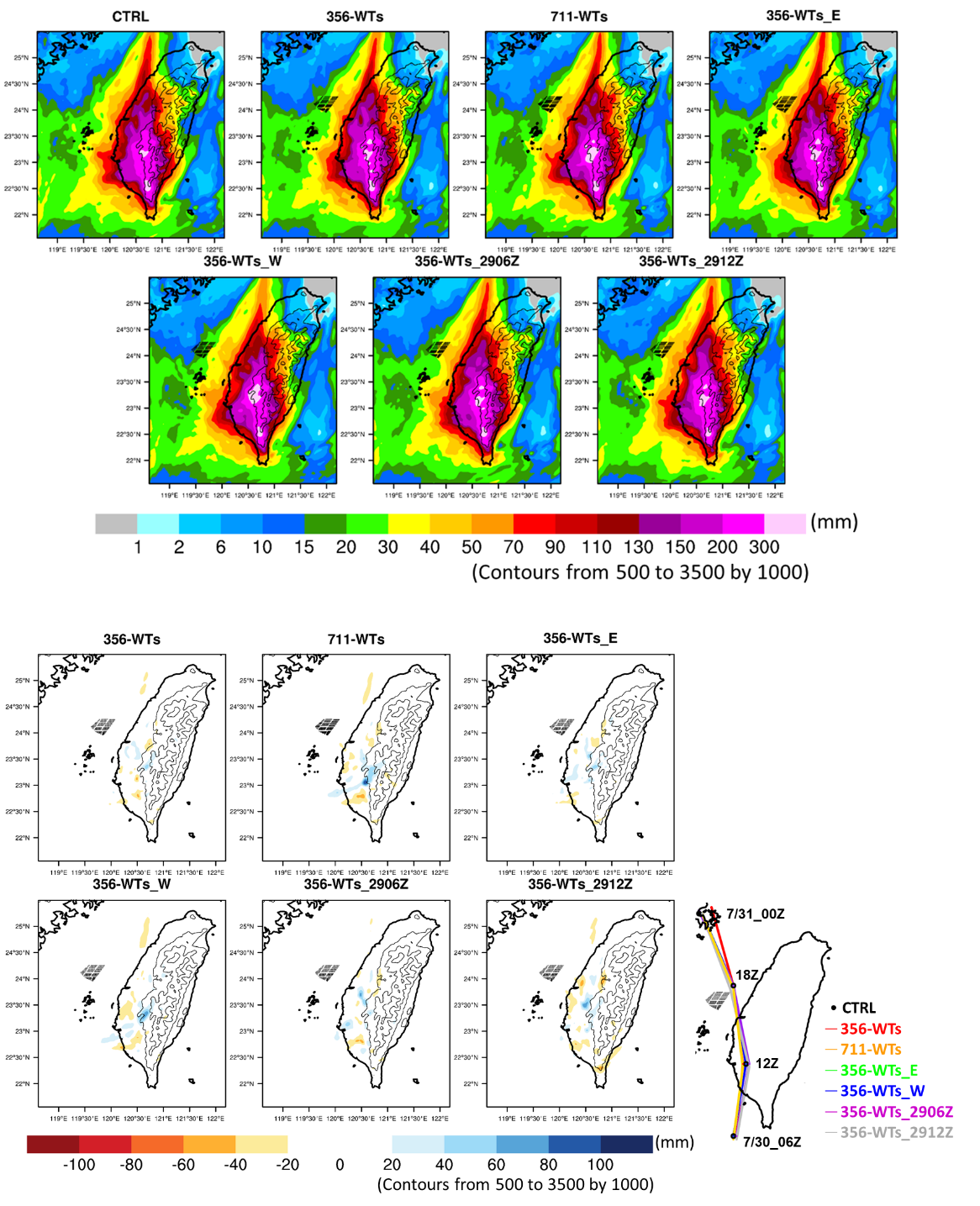
Figure S1: upper panel: 12-hour accumulated precipitation (mm) in cases CTRL and sensitivity studies (listed in table 1) over Taiwan during 12:00–23:00 UTC on July 30, 2017. Contours indicate the terrain height (m). Bottom panel: Differences in accumulation rainfall between sensitivity cases (listed in table 1) and CTRL simulation during 12:00–23:00 UTC on July 30, 2017. Contours indicate the terrain height (m).

Maps and plots produced using NCAR Command Language (NCL) version 6.6.2[34].
